# Dengue Outbreak in Key West, Florida, USA, 2009

**DOI:** 10.3201/eid1801.110130

**Published:** 2012-01

**Authors:** Elizabeth G. Radke, Christopher J. Gregory, Kristina W. Kintziger, Erin K. Sauber-Schatz, Elizabeth A. Hunsperger, Glen R. Gallagher, Jean M. Barber, Brad J. Biggerstaff, Danielle R. Stanek, Kay M. Tomashek, Carina G.M. Blackmore

**Affiliations:** Florida Department of Health, Tallahassee, Florida, USA (E.G. Radke, K.W. Kintziger, E.K. Sauber-Schatz, D.R. Stanek, C.G.M. Blackmore);; Centers for Disease Control and Prevention, San Juan, Puerto Rico (C.J. Gregory, E.A. Hunsperger, G.R. Gallagher, K.M. Tomashek);; Centers for Disease Control and Prevention, Atlanta, Georgia, USA (E.K. Sauber-Schatz);; Monroe County Health Department, Key West, Florida, USA (J.M. Barber);; Centers for Disease Control and Prevention, Fort Collins, CO, USA (B.J. Biggerstaff)

**Keywords:** dengue, Aedes aegypti, seroprevalence, outbreak, viruses, vector-borne disease, Key West, Florida

## Abstract

After 3 dengue cases were acquired in Key West, Florida, we conducted a serosurvey to determine the scope of the outbreak. Thirteen residents showed recent infection (infection rate 5%; 90% CI 2%–8%), demonstrating the reemergence of dengue in Florida. Increased awareness of dengue among health care providers is needed.

Dengue is the most common mosquito-borne viral infection worldwide (*1*); however, it has been eliminated from the continental United States. No locally acquired cases have been reported outside the Texas–Mexico border (*2**,**3*) in >60 years, despite global increases in incidence and severity. Concern exists that dengue virus (DENV) may be reintroduced (*4*) because it is the most frequent cause of febrile illness among travelers returning from the Caribbean, South America, and Asia (*5*). Immigrants and visitors from dengue-endemic countries also provide opportunity for its reintroduction (*6*).

In September 2009, the Florida Department of Health (FDOH) was notified that a person with suspected dengue had recently traveled to Key West, Florida. The Centers for Disease Control and Prevention (CDC) (Atlanta, GA, USA) confirmed the diagnosis. Subsequently, dengue was confirmed in 2 Key West residents without a history of recent travel. FDOH and CDC conducted an investigation to determine size and scope of the outbreak. Cases identified through passive surveillance were reported (*7*).

## The Study

Key West is a tourist destination with >2 million visitors annually (*8*). Old Town, the area with reported cases, has a population of 19,846 (*9*). *Aedes aegypti* mosquitoes, the usual vectors of dengue, are widespread in Key West, but *Ae. albopictus* mosquitoes are uncommon.

In September 2009, we surveyed Old Town residents to estimate the infection rate and identify risk factors using stratified, 1-stage cluster sampling to randomly select 911 (15%) households within 1 km of the residence of the index case-patient. The area around the residences of the index case-patients was divided into 3 strata: strata 1 and 2 (within 200 m of each case-patient) and stratum 3 (201–1,000 m). Investigators asked household members (>5 years of age) for a blood sample and information on recent illness, travel, foreign residence, and risk factors for dengue. One adult per household completed a questionnaire concerning the household. Investigators revisited unresponsive households 3 times unless the homes were empty.

Serum specimens were screened by ELISA for dengue-specific IgM and IgG (*10**,**11*). IgG-positive samples were tested by plaque reduction neutralization test with 90% cutoff (PRNT_90_) against DENV serotypes 1–4 and West Nile virus (*12*). A >4-fold difference in titer between viruses was used to identify the infecting virus. Participants reporting febrile illness within a week were tested by reverse transcription PCR for DENV and West Nile virus and by nonstructural protein 1 ELISA for DENV antigen.

We classified participants with laboratory-positive DENV infection as follows: acute, if positive with reverse transcription PCR or nonstructural protein 1 ELISA; recent, if IgM-positive ELISA and PRNT_90_ results were consistent with DENV infection; and presumptive recent, if they had dengue-like illness within 3 months, IgG-positive ELISA, and PRNT_90_ results consistent with DENV infection. We classified participants as having previous DENV infection if they had IgG-positive ELISA and PRNT_90_ results without recent febrile illness.

We weighted responses to account for sampling design using different probabilities of inclusion across strata and within-household participation rates, allowing for population inference (*13*). CIs accounted for sampling design and finite population correction factors. We used weighted logistic regression to assess risk factors for infection, and resulting inferences accounted for sampling design. Tests were performed in SAS version 9.2 (SAS Institute, Cary, NC, USA); p = 0.10 was significant.

Informed consent was obtained from all participants >18 years of age. Assent from the minor and informed consent from a parent were obtained for minors.

Of 911 selected households, 200 (22%) had been vacated, 387 (42%) did not have a resident at home, and 324 (36%) had a resident contacted; 170 (52%) households and 240 persons participated. Median age was 53 years (range 15–95), slightly older than the median population (41 years) of Old Town (*14*). Most participants were non-Hispanic white (78%) and male (58%), similar to Old Town’s population (*14*). Forty-three (18%) had lived in dengue-endemic countries, and most (148, 62%) had previously traveled to dengue-endemic areas.

**Figure 1:**
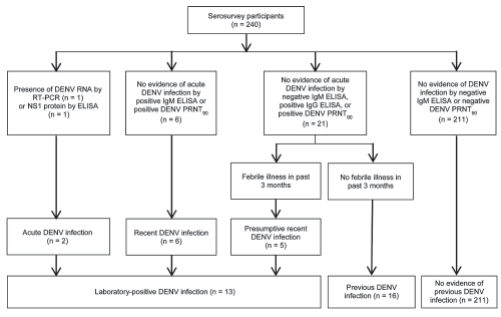
Case classification of 240 household survey participants Key West, Florida, USA, September 2009. DENV, dengue virus; RT-PCR, reverse transcription PCR; NS, nonstructural; PRNT_90_, plaque reduction neutralization test with 90% cutoff.

**Figure 2:**
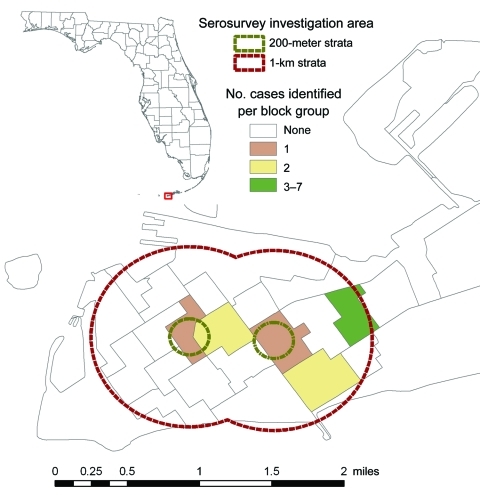
Locations of laboratory-positive cases of dengue in Key West, Florida, USA, in household survey, September 2009.

Thirteen (5%; 90% CI 2%–8%) participants had laboratory-positive DENV infections (2 acute, 6 recent, 5 presumptive) (Figure 1). Without including presumptive infections, the rate was 3% (90% CI 1%–4%). Acute infections were confirmed as DENV-1. Two samples from those persons with presumptive infections had antibodies against DENV-1 by PRNT_90_; samples from 3 persons were cross-reactive with multiple DENV serotypes. Infection rates were 4% in strata 1 and 3 (90% CI 0%–11% and 0%–6%, respectively) and 17% in stratum 2 (90% CI 0%–33%) (Figure 2). Sixteen (6%; 90% CI 3%–8%) participants had previous DENV infections, with antibodies against multiple serotypes (Figure 1). Seventy participants had IgG-positive ELISA results, but confirmatory testing did not show DENV infection.

Persons who kept windows open >50% of the time, lived in a household with >50% of the yard covered with vegetation, had a bird bath, or reported receiving mosquito bites at work were more likely to be infected with DENV (Table). Persons who used air conditioning >50% of the time, emptied containers of standing water weekly, and used *N,N-*diethyl-*m*-toluamide–containing repellents were less likely to be infected. African Americans were more likely (19%) to be infected than whites (4%; p = 0.09).

**Table:** Risk factors associated with laboratory-positive dengue virus infection among residents of Key West, Florida, USA, according to household survey, September 2009*

Variable	No. (%)† persons with infection, n = 13	No. (%) persons without recent infection, n = 227	Crude OR (90% CI)‡
Bird bath in yard	5 (41)	26 (11)	5.6 (1.5–21.3)
Windows open >50% of the time	5 (41)	37 (15)	3.9 (1.1–14.0)
Vegetation covers >50% of yard	8 (59)	61 (30)	3.4 (1.0–11.2)
Outside in evenings	11 (86)	149 (67)	3.1 (1.0–9.5)
Bitten by mosquito at work/school	5 (32)	28 (14)	3.0 (1.1–8.2)
Uses repellent containing DEET	3 (20)	98 (41)	0.4 (0.1– 0.9)
Uses mosquito bite prevention measures	4 (26)	119 (52)	0.3 (0.1–0.7)
Air conditioning on >50% of time	6 (37)	170 (75)	0.2 (0.1– 0.6)
Traveled outside Florida in past 3 mo	2 (12)	93 (38)	0.2 (0.1–0.9)
Empties water from containers regularly	1 (6)	79 (36)	0.1 (0.0–0.7)

*OR, odds ratio; DEET, *N,N-*diethyl-*m*-toluamide. †Weighted percentages are reported, reflecting the stratified, 1-stage cluster sampling design. Responses were weighted to account for the different probabilities of household inclusion across strata, within-household participation rates, and interhousehold clustering of infections. ‡Significance level, p = 0.10. Weighted logistic regression models were used to assess risk factors for recent infection, and CIs were based on the modeling accounted for the sampling design.

## Conclusions

Approximately 3%–5% of Old Town residents were infected with DENV during July–September 2009, and surveillance identified new cases through October (*7*). Additionally, 63 cases, caused by the same strain of DENV-1, were reported there in 2010. All infections in Key West seem to have been locally acquired. Our findings that less frequent use of air conditioning, leaving windows open, and yard vegetation were risk factors for infection agree with results of previous dengue studies (*2**,**3**,**14*).

A specific route and time of introduction of dengue to Key West cannot be identified. The island has many tourists from dengue-endemic countries and a well-traveled population. Because the clinical features of dengue are often nonspecific, transmission was likely ongoing before the index case-patient’s condition was diagnosed and FDOH was notified. Without diagnosis of this case, the outbreak may not have been recognized. Efforts to inform local healthcare providers about the identification, diagnosis, and clinical management of dengue are ongoing.

A useful discovery was the evidence of previous infections with other serotypes among residents, which indicates that a segment of the population is at risk for secondary infection, which is associated with severe dengue (*15*). The survey population was well traveled, placing them at risk of acquiring severe disease during travel to dengue-endemic countries or of different dengue serotypes being introduced into Key West. Other risk factors include the age, immune status, and genetic background of the infected person (*15*).

This investigation was performed quickly after outbreak identification while transmission was ongoing, which allowed acute infections to be identified. Study limitations included the circumstances that many households could not be contacted and many residents refused to participate. We attempted to minimize participation bias through our sampling scheme. Also, presumptive infections may have been misclassified, which makes the 3% infection rate more likely to be accurate. Other misclassification because of prior flavivirus infection is possible, but was minimized by careful interpretation of laboratory results.

This outbreak and its continuation into 2010 demonstrate the potential for reemergence of dengue in subtropical areas of the United States where *Ae. aegypti* mosquitoes are present. Awareness among healthcare providers should be increased for optimal patient management and to limit outbreaks. Minimizing the effects of future dengue epidemics depends on personal protection against mosquitoes, mosquito control, early diagnosis, appropriate testing, and prompt reporting of suspected cases to public health authorities.
